# Improving Functional Communication Outcomes in Post-Stroke Aphasia via Telepractice: An Alternative Service Delivery Model for Underserved Populations

**DOI:** 10.5195/ijt.2022.6531

**Published:** 2022-12-13

**Authors:** Portia Carr, Dana Moser, Shana Williamson, Greg Robinson, Stephen Kintz

**Affiliations:** 1 University of Arkansas for Medical Sciences, Little Rock, Arkansas, USA; 2 University of Arkansas at Little Rock, Little Rock, Arkansas, USA

**Keywords:** Aphasia, Conversational script training, Oral reading for language in aphasia, Telepractice

## Abstract

Many persons with aphasia (PWA) have limited access to speech-language treatment (SLT) due to limited funding, speech-language pathologist shortages, geographical barriers, physical disabilities, transportation barriers, and the COVID-19 pandemic. The purpose of this study was to determine if telepractice is an effective and feasible service delivery model for PWA. Ten PWA completed 8 hours of remote treatment over 4 weeks. Synchronous telepractice sessions employed Oral Reading for Language in Aphasia (ORLA) and Conversational Script Training (CST). Pre- and post-assessment outcome measures included the Communication Activities of Daily Living-3 (CADL-3) and the Communication Confidence Rating Scale for Aphasia (CCRSA). Participants completed a telepractice satisfaction survey following post-assessment. All participants demonstrated improvements in CCRSA scores, total words produced correctly on trained CST stimuli, and total words produced correctly on trained ORLA stimuli. No differences were noted in CADL-3 scores. All participants were highly satisfied with telepractice as a service delivery model.

Stroke is a leading cause of serious long-term adult disabilities. Stroke health care services, medications to treat stroke, and missed days from work cost an estimated $52.8 billion annually (Tsao et al., 2022). According to the National Stroke Association, 2 million people in the United States have strokes each year, and 38% of stroke survivors are diagnosed with aphasia (Thomas, 2021). Aphasia is an acquired language disorder following brain injury (e.g., stroke or head injury). Approximately 2.5 to 4 million people in the United States are currently living with aphasia (Simmons-Mackie, 2018).

Aphasia may impair verbal expression and comprehension of language, as well as reading and writing. The prognosis of persons with aphasia (PWA) varies widely across individuals. The greatest amount of spontaneous recovery is seen within the first 3-months post-stroke and then decreases with significantly less recovery after the first year (Munsell et al., 2020). PWA often experience negative long-term impacts on their relationships, occupation, mental health, socialization, independence, and quality of life (Choi et al., 2016). One of the most concerning long-term impacts of PWA is expressive language impairments in functional everyday life settings. PWA often report difficulty talking on the phone, participating in meetings, ordering off menus, interacting at the bank, and communicating in conversations about complex themes (Rhodes & Isaki, 2018). Many PWA in metropolitan and rural areas have limited access to speech therapy upon discharge from hospitals, rehabilitation facilities, and skilled nursing facilities. Additionally, PWA in rural areas are disproportionally impacted due to staff shortages of SLPs and high turnover rates (Jacobs et al., 2021).

Speech-language therapy (SLT) is a standard component of the functional recovery process for individuals with communication deficits after a stroke. Extensive research supports the fact that PWA continue to improve their language and communication abilities when treatment is continued well beyond post-stroke onset (Hall et al., 2013; Lavoie et al., 2017; Molini-Avejonas et al., 2015; Munsell et al., 2020; Zheng et al., 2016). The management of aphasia is often an ongoing longterm process due to residual communication impairment and psychosocial impacts. SLT is an integral component of the acute and chronic stages of recovery, but many PWA have a limited number of therapy sessions due to lack of insurance reimbursement, transportation barriers, health care disparities, speech-language pathologist (SLP) shortages, case-load capacities, and geographical barriers (Choi et al., 2016). Persistent gaps in equitable speech-language pathology services is an ongoing challenge for PWA residing in medically underserved areas (Choi et al., 2016; Hakim et al., 1998).

Additionally, the Coronavirus of 2019 (COVID-19) further reduced access to SLT due to patients' reluctance to attend in-person therapy, staff shortages, delayed home health referrals, outpatient clinic closures and reductions in operating hours (Gustavson et al., 2021). The pandemic has exacerbated isolation, stress, mental health, and physical, and cognitive issues among individuals with disabilities, who are already more likely to be economically disadvantaged and have inequitable access to health care (Ertelt, 2020; Gustavson et al., 2021). Furthermore, the pandemic has further highlighted the disproportionate disparities among individuals with disabilities including PWA (Ertelt, 2020). Telepractice may be an effective alternative service delivery model to improve access to SLT for PWA.

Ultimately, SLT is about improving quality of life and life participation. Studies have found positive effects regarding generalization of outcomes and patient satisfaction when SLT is delivered in a person's home setting versus a clinic, especially when treatment objectives are specific to the patient's everyday life (Theodoros, 2014). Telepractice provides a unique opportunity to target the patient's individualized functional communication goals in their natural environment by implementing evidence-based treatments that correspond with the commonly used Life Participation Approach to Aphasia (LPAA). LPAA is a patient-driven service-delivery approach that involves the PWA in the decision-making process for developing treatment goals for re-integration in society, thus improving their overall quality of life. LPAA focuses on the reasons to communicate in addition to the rehabilitation of functional communication (Chapey et al., 2000). LPAA and telepractice are both in alignment with the World Health Organization framework which emphasizes the importance of a person functioning within the context of their natural environment (WHO, 2001).

In order to capitalize on personally relevant stimuli, the current study employed two evidence-based aphasia treatment interventions that could improve the person's functional communication during life participation activities: Conversational Script Training (CST) and Oral Reading for Language in Aphasia (ORLA) TM. CST utilizes personalized scripts that can be used in everyday life and has a relatively transparent application for functional communication and the LPAA. Conversational Script Training is a functional aphasia treatment in which the participant verbally produces a personally relevant script in a hierarchy of repetition, choral reading, and independent production. Scripts are intended to guide and facilitate participants' conversations and actions in social situations (Holland et al., 2002; Youmans et al., 2011). Script training is based on the theory of automatization which suggests that automaticity is achieved through repeated exposure and practice (Holland et al., 2002).

ORLA is an aphasia treatment in which the PWA reads paragraphs and sentences systematically in unison with the clinician and then independently. It was originally developed to target reading comprehension by providing practice through semantic and phonological pathways. While not as directly functional, the personally relevant passages used in the ORLA protocol have the potential to strengthen semantic and phonological pathways while also impacting reading and prosody. Thus, it is reasonable to hypothesize that ORLA could facilitate improved functional communication overall.

Previous studies have investigated the efficacy of CST and ORLA with PWA when they were administered separately via in-person SLT, asynchronous telepractice, or synchronous telepractice (Cherney, 2004; Cherney et al., 2011, 2015; Holland et al., 2002; Rhodes & Isaki, 2018; Youmans et al., 2011). Prior studies have found ORLA to be effective for improvements in other modalities including auditory comprehension, verbal expression, and written expression in fluent and non-fluent aphasia (Cherney et al., 1986; Cherney, 1995, 2004; Cherney, Babbitt, Kim et al., 2011). CST has been found to be effective when delivered via in-person SLT and via telepractice (Cherney et al., 2015; Holland et al., 2002; Rhodes & Isaki, 2018; Youmans et al., 2011). The current study investigated the effects of CST and ORLA during synchronous telepractice sessions to determine if PWA would demonstrate improvements in functional communication.

## Telepractice in PWA

The following literature review provides a summary of previous studies investigating the effectiveness of aphasia evaluation and intervention administered to PWA using telepractice. Several studies have suggested that telepractice services for persons with chronic aphasia yields comparable outcomes to in-person SLT. Theodoros et al. (2008) simultaneously assessed 32 PWA dispersed among two randomized groups via telepractice and in-person delivery. Participants were administered the short form of the Boston Diagnostic Aphasia Exam (BDAE-3; Goodglass & Kaplan, 1983), Boston Naming Test 2^nd^ edition (BNT; Kaplan et al., 2001), and a participant satisfaction questionnaire. The in-person group was assessed at the Queensland University Clinic. The telepractice group was assessed while the SLP was at the university clinic and the participant were at a local hospital. Assessment scores were compared with the Wilcoxon signed rank test which did not indicate any significant differences between the groups. There was a 93% satisfaction rate among the telepractice group. The results indicate that assessment utilizing telepractice is a comparable, effective, and feasible alternative to in-person assessment for PWA (Theodoros et al., 2008).

A study by Guo et al. (2017) investigated the reliability of an iPad-based aphasia assessment application called Access2Aphasia ™ and the use of supported conversation techniques. Thirty PWA of varying severity levels were randomized into an in-person assessment and Access2Aphasia application group. Participants were administered the Spoken Word–Picture Matching and Spoken Word Naming subtests of the Psycholinguistic Assessment of Language Processing Activities (PALPA; Kay et al., 2001) and the Assessment of Living with Aphasia (ALA; Simmons-Mackie et al., 2013) to allow outcomes to be captured across the International Classification of Functioning (ICF) domains. All participants and SLPs in the telepractice group were given a satisfaction questionnaire. The telepractice group was assessed in their home while the SLP was in a different location. The in-person group was also assessed while in their homes with the SLP present. Kappa statistics indicated a 99% agreement rate between online and in-person assessment. PWA and SLPs were both satisfied with the telepractice Access2Aphasia approach.

Dekhtyar et al. (2020) examined the validity of telepractice administration of the Western Aphasia Battery-Revised (WAB-R; Kertesz, 2007). Twenty PWA were counterbalanced with all participants completing the WAB-R in-person and via videoconference. The WAB-R was administered with specific pre-determined telepractice modifications. The researchers reported satisfaction with both platforms, but stated Zoom was more user friendly due to the ease of sharing documents. Three of the twenty participants preferred to complete the assessments in the clinic instead of in their homes. The researchers provided a laptop in a separate therapy room and simulated the same conditions as the participants who completed the assessments in their homes. The results were analyzed with intra-class correlations and paired-samples t-tests which revealed no significant differences between the groups. The satisfaction survey revealed high satisfaction with 85% of participants indicating no preference of one administration method over the other (Dekhtyar et al., 2020).

Hall et al. (2013) conducted a systematic review of telepractice assessment and treatment of individuals with aphasia. They reviewed 10 studies that all confirmed the reliability and feasibility of telepractice assessment. There was no difference between in-person or telepractice assessment scores for any of the review studies. The severity of the aphasia did not influence the results. The review revealed several advantages and disadvantages of telepractice assessment. Advantages were as follows: improved alertness to stimuli, reduced cost of travel, decreased cost to treatment, more effective use of time, successful delivery of services to those who would otherwise be unable to receive services due geographical location, and improved attendance and adherence to intervention protocols. Disadvantages were as follows: difficulty sustaining telephone and/internet connections, audio/video delay, reduced quality of visual cues and stimuli than in traditional settings, and client concerns regarding privacy. However, technology improvements within recent years may counteract some of the disadvantages.

Multiple studies have demonstrated telehealth to be an effective and feasible delivery modality for providing increased access to SLT, continuity of care, and reducing health care costs. Molini-Avejonas et al. (2015) conducted an international review to investigate telehealth application in speech-language and hearing sciences. They reviewed 103 papers that focused on several domains including: hearing (32.1%), speech (19.4%), language (16.5%), voice (8.7%), swallowing (5.8%), multiple areas (13.6%) and others (3.9%). Most studies concluded that telehealth had advantages over the non-telehealth alternative approach (85.5%) and 13.6% reported that it was unclear whether the telehealth procedure had advantages. The primary advantage reported from the review studies was improved access to SLT (80.6%). Among the language studies, aphasia was the most investigated disorder (41.2%). The most prevalent purposes of the aphasia studies were to evaluate satisfaction with telehealth (64.7%), to assess the use of software via remote diagnosis (64.7%), followed by comparing results from in-person and telehealth groups (58.8%). The majority of the studies revealed positive outcomes of telehealth and indicated it is comparable to in-person intervention (Molini-Avejonas et al., 2015).

Lavoie et al. (2017) conducted a systematic review with 23 studies to investigate the effectiveness of treatments delivered by technology in the management of post-stroke anomia. They assessed the following primary outcomes: (1) improvement in naming skills; (2) generalization to untreated items; and (3) impact of therapy on functional communication. All studies confirmed the effectiveness of anomia therapy provided by technology. The review studies found improvements in naming trained items, but generalization to untreated items and everyday life communication was inconsistent. The authors noted that generalization to untrained items and everyday life communication is inconsistent in both telepractice and traditional in-person SLT.

Macoir et al. (2017) investigated the effectiveness of synchronous telepractice using Oralys TeleTherapy TM software which is based on the Promoting Aphasics' Communicative Effectiveness (PACE) approach. Twenty participants with chronic post-stroke aphasia participated in pre-test and post-test design. They received nine speech therapy sessions over a three-week period. They reported improvements in functional communication as evidenced by: (1) an increase in communication effectiveness, reflecting significantly improved autonomy in functional communication; (2) a decrease in communication exchange duration, meaning that the treatment made communication faster and more efficient; (3) a decrease in the number of communication acts, meaning that, after treatment, less information was needed to be efficiently understood by the communication partner; and (4) an increase in the number of different communication strategies used, meaning that the treatment fostered the use of a variety of alternative communication modes. A limitation of the study was the lack of functional outcome measures to determine the generalization of therapy gains to functional communication during activities of daily living.

Zheng et al. (2016) conducted a systematic review comparing the effectiveness of synchronous technology-based SLT compared to no therapy and compared to in-person therapy. They reviewed seven studies to assess the effectiveness of computer programs targeting different areas of language and concluded that computer therapy was effective in comparison to no therapy. The three studies that compared computer-delivered therapy to in-person therapy reported improvements with no significant differences between the groups, indicating computer-delivered therapy could be as effective as clinician-delivered therapy for individuals with chronic aphasia.

Woolf et al. (2016) conducted a quasi-randomized study to examine the treatment effectiveness, treatment fidelity, and compliance and satisfaction with technology of PWA receiving remote therapy using FaceTime versus in-person therapy. Twenty-one PWA were assigned to either FaceTime intervention provided from a university lab, FaceTime intervention provided from a clinical site, in-person therapy, or a control group with conversations held remotely. PWA received picture naming therapy and were required to choose their communication partner. All groups received picture naming therapy for one hour twice a week for four weeks. The primary outcome measures included the spoken picture naming, semantic memory, and recognition memory subtests of the Comprehensive Aphasia Test (CAT; Swinburn et al., 2004). The secondary outcome measure was the assessment of naming in conversation. The participants engaged in a 10-minute conversation that was analyzed according to the proportion of substantive turns, mean number of content words per turn, and the mean number of nouns per turn. All groups who received therapy improved picture naming abilities significantly more than the control group. There were no significant differences between the therapy groups in regard to naming in conversation. The authors attributed the lack of improvements in conversation to the treatment being focused on single picture naming instead of conversational tasks.

Steele et al. (2015) examined the feasibility of combining individual and group teletherapy with online-language exercises. Nine participants with chronic aphasia received three hours of remote individual therapy in their homes and eighteen hours of remote group therapy at a clinic. They completed online language exercises with the TalkPath TM application between therapy sessions. Pre-test and post-test measures included the Western Aphasia Battery-Revised (WAB-R; Kertesz, 2007), a portion of the Communicative Effectiveness Index (CETI; Lomas et al., 1989), ASHA National Outcome Measurement System (NOMS), and the RIC Communication Confidence Rating Scale for Aphasia (RIC-CCRSA; Cherney, Babbitt, Semik, & Heinemann, 2011). The participants were also given a satisfaction survey at the end of the study. Pre-treatment and post-treatment means were calculated and compared. Matched *t-tests* were used to determine the significance of improvements following treatment. Analysis of scores revealed improvements in all measures except the RIC-CCRSA. The authors acknowledged the ongoing challenges regarding the acceptance of teletherapy as an alternative cost-effective service delivery model for persons with chronic aphasia.

Simic et al. (2016) investigated the usability of synchronous telepractice for improving naming deficits in persons with chronic post-stroke aphasia using the PhonoCom™ application. The usability of the PhonoCom application was assessed based on effectiveness, efficiency, and satisfaction. Six participants with mild-moderate aphasia were split into remote and in-person groups, which received phonological component analysis naming therapy. The results were analyzed via a Wilcoxon signed-rank test which revealed naming improvements in both the telepractice and in-person groups. The findings indicated improvements in naming deficits with high participant satisfaction.

Weidner and Lowman (2020) examined the feasibility and efficacy of speech-language pathology services by reviewing 31 adult telepractice service articles published between 2014–2019. The studies investigated feasibility, efficacy, diagnostic accuracy, and non-inferiority of SLT in-person and telehealth services across various adult populations. The most investigated disorder was aphasia (48%); followed by Parkinson's disease (16%). The results supported the efficacy and feasibility of telepractice in speech-language pathology for adults.

The studies referenced above suggest there is no difference between the functional outcomes of aphasia intervention delivered in person and via telepractice. However, more evidence is needed to support telepractice as standard practice. Moreover, a limited number of telepractice studies have utilized functional communication assessments to measure the efficacy of telepractice with PWA. Research indicates PWA experience better outcomes when goals are salient and personally relevant (Cherney et al., 2015). Therefore, aphasia assessment and goal planning should consider the PWA's life participation and quality of life. However, most studies have been limited to the use of impairment-based assessments.

## Purpose and Specific Aims

The purpose of this study was to determine if telepractice is an effective and feasible service delivery model for PWA. The study included the following specific aims:

1. To determine if PWA would demonstrate improvements in functional communication outcome scores after telepractice intervention as measured by language assessment scores.
2. To determine if PWA would demonstrate improvements in functional communication as measured by performance on treated functional communication measures.
3. To determine the feasibility of telepractice for PWA as measured by a telepractice satisfaction survey.

## Methods

### Participants

In order to be included in the study, participants were required to meet the following criteria: (a) Age range between 40 and 89 years; (b) Native English speaker per self-report; (c) mild-moderate aphasia as determined by an aphasia quotient score ranging from 51.0 to 93.8 on the Western Aphasia Battery-Revised (WAB-R; Kertesz, 2007). Exclusionary criteria included: (a) uncorrectable hearing or vision per informal screening measures; (b) less than 6-months post-stroke per self-report; and (c) severe auditory comprehension deficits.

Twelve PWA were screened and initially agreed to participate in the study. One PWA was excluded because her WAB-R quotient was in the severe range. Another participant completed the telepractice screening process but decided not to enroll due to concerns regarding the usability of the Zoom Healthcare TM videoconferencing system. Ultimately, ten participants between the ages of 43 and 67 years enrolled in the study. Two of the ten participants received telepractice at the University of Arkansas for Medical Sciences Speech-Language, and Hearing Clinic due to lack of access to a sufficient device (i.e., desktop, laptop, tablet) and internet service. In an effort to replicate the condition of the other study participants, the two participants were positioned in a separate room from the therapist. The participants' demographics are displayed in Table 1.

**Table 1:** Demographic Data and WAB-R Aphasia Quotient (AQ) Scores

Participant	Age	Gender	Race	Education	Years Post-stroke	WAB-R AQ
1	60	F	W	16	5	71.8
2	43	F	B	12	12	74.3
3	47	F	B	12	2	74.4
4	64	F	B	14	6	79.2
5	51	F	B	16	14	81.4
6	67	M	W	16	2	85.7
7	60	M	W	12	5	87.2
8	46	M	W	15	1	90.6
9	53	M	B	12	0.75	90.8
10	63	F	B	14	1	90.0

### Pre- and Post-Assessments

All participants completed a series of assessments, administered remotely to measure their language abilities. A telespeech hearing screening and the Western Aphasia Battery - Revised (WAB-R; Kertesz, 2007) were administered as qualifying measures prior to intervention to determine eligibility and obtain a clinical profile for aphasia severity. Eligible participants completed pre-and post-assessments including the Communication Activities of Daily Living-3 and the Communication Confidence Rating Scale for Aphasia to measure changes in performance following intervention. The Communication Activities of Daily Living-3 (CADL-3 is a standardized measure of functional communication skills which assesses reading/writing, using numbers, social interactions, contextual communication, non-verbal communication, sequential relationships, humor, metaphor, absurdity, and internet basics (Holland et al., 2018). The Communication Confidence Rating Scale for Aphasia (CCRSA) is a self-rating measure of the PWA's perception and confidence of communication skills (Cherney, Babbitt, Semik, & Heinemann, 2011). A telepractice satisfaction survey was administered during the post-assessment phase. The telepractice satisfaction survey was created by modifying the Telehealth Usability Questionnaire (TUQ). The TUQ was developed to determine the usability of telehealth using a Likert scale to rate factors including usefulness, ease of use and learnability, interface quality, interaction quality, reliability, and satisfaction (Parmanto et al., 2016).

The WAB-R and CADL-3 were administered with telepractice assessment modifications. The CADL-3 and CCRSA pre- and-post assessments were administered remotely over two to three sessions lasting 60 minutes each. The participants completed the telepractice satisfaction survey at the end of the post-assessment phase as a measure of patients' satisfaction with telepractice as a service delivery model.

Each participant underwent four weeks of remote treatment consisting of 60-minute sessions twice weekly (two days/week for four weeks = eight sessions). Each participant completed one training session, four to five assessment sessions (e.g., pre-/post-assessment), and eight treatment sessions for a total of 12–14 sessions. Synchronous real-time telepractice sessions were conducted using the Zoom videoconferencing platform. This platform was selected due to the perceived ease of use and cost efficiency. The researchers used a licensed Health Insurance Portability and Accountability Act (HIPAA) compliant Zoom for Healthcare account with a Business Associates Agreement (BAA) for all sessions. Each participant received a maximum of one hour of remote training for using both the computer and the video conferencing website prior to the first assessment session. In addition, they received a training manual and cheat sheet to assist with logging on and troubleshooting. Speech-language treatment (SLT) consisted of evidenced-based treatments that correspond to the Life Participation Approach to Aphasia (LPAA). Each participant received approximately 30 minutes of Oral Reading for Language in Aphasia (ORLA) and 30 minutes of Conversational Script Training (CST) during each session. Both treatment interventions were modified to be administered via the telepractice modality.

**Figure 1:**
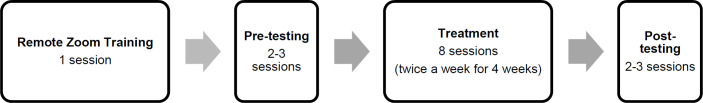
Study Timeline

### Treatment

#### Conversational Script Training (CST)

The participants targeted CST using two personally relevant scripts that were developed by the PWA and the clinician before the first treatment session. Script topics were determined during a one-hour interview with the participant prior to the first treatment session. The script topics were based on the PWA's individualized interests, hobbies, values, experiences, and lifestyles. The most common script topics included ordering food in a restaurant, making phone calls, and asking salesclerks for specific items in the store. Once the scripts were developed, the clinician and PWA modified the scripts to use vocabulary and syntax that were natural for the PWA. Then baseline data for the total words produced were collected for both scripts. The criteria for script mastery were 90% accuracy on words produced correctly for the entire script across two consecutive sessions. The second script was targeted upon mastery of the first script.

#### Oral Reading for Language in Aphasia (ORLA)

In the current study, the participants targeted ORLA using two personally relevant reading passages. The reading passage topics were determined via a one-hour interview with the participants prior to the first treatment session. Passages were based on the PWA's individualized interests, hobbies, and values. Goals were developed for each participant as determined by the baseline data for each reading level and the baseline accuracy of the total words produced. Prior to the first treatment session, each participant was presented with a reading passage for each reading level (1–4) to determine their baseline reading-passage level. Reading levels were as follows: Level 1—simple 3- to 5-word sentences at a first-grade reading level; Level 2—8 to 12 words that may be single sentences or two short sentences, at a third-grade reading level; Level 3—15 to 30 words, divided into two to three sentences, at a sixth-grade reading level; Level 4—50 to 100 words comprising a four- to six-sentence simple paragraph, also at a sixth-grade reading level (Cherney, et al., 1986; Cherney, 2004). If the participant achieved less than 90% accuracy for the total words produced on the baseline on a particular reading level, they began at that respective reading level. If the baseline for the total words produced was 80% or lower, their goal was 90% accuracy. If the baseline was 80% accuracy or higher, their goal was 95% accuracy. After the first script was mastered, a baseline for the total words produced was obtained for the previously targeted reading level as well as the next reading level. If the baseline was less than 90% accuracy, they continued at the previously targeted reading level. If the baseline was higher than 90% accuracy, they proceeded to the next respective reading level. If the participants had a baseline over 90% accuracy for level 4, the goal was targeted at level 4 while using more semantically complex words and multiple paragraphs.

### Data Analysis

Treatment-related data was collected for PWA during each session. Specific data collected for ORLA and CST treatment included improved accuracy of words produced correctly. The treatment data were analyzed to compare the pre-treatment baseline performance from the first treatment session to the post-treatment performance from the last treatment session for each trained ORLA passage and trained CST script. Pre- and post-treatment scores from the CADL-3, CCRSA, and treatment targets were analyzed to investigate the change in severity level across the two time points. The Wilcoxon Signed Rank test was conducted to determine the relative effectiveness of SLT delivered via telepractice. Cohen's r was used to compute the effect size of the gains in treatment target performance from baseline to the performance from the last treatment session. The telepractice satisfaction survey was analyzed by determining the mean percentage of the Likert scale responses ranging from (1–5) for each participant.

### Interrater Reliability

Percent agreement was used to determine the degree to which two raters reported the same values. A second researcher independently analyzed five random assessment sessions and four random treatment sessions using the same scoring system as the original rater. There was a very high level of exact agreement (*M*= 98.08) between the two raters on the assessments. There was also a very high level of exact agreement between the two raters on the total words produced correctly during CST (*M* = 95.7) and the total words produced correctly during ORLA (*M*= 98.18). The mean exact agreement for CST and ORLA intervention combined was 97.07.

## Results

### Generalized Communication Measures

**Figure 2:**
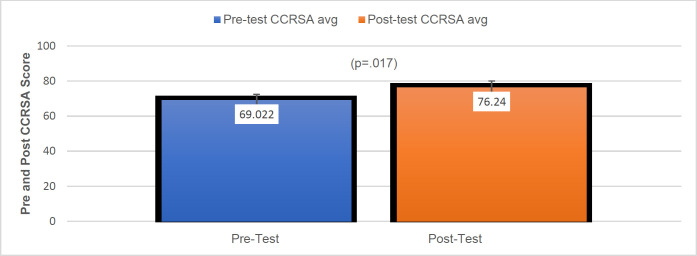
Group Means for CCRSA Pre-Test and Post-Test Scores

The first aim of this study was to determine if PWA would demonstrate improvements in functional communication outcome scores after telepractice intervention as measured by language assessment scores. Two generalized communication measures were used to address this aim: The CCRSA and the CADL-3. A Wilcoxon signed rank test revealed statistically significant improvements in the pre-and post-test CCRSA scores with a large effect size *T*= 51.00, *z* = 2.40, *p* = .017, *r* =.53 (see Figure 2). The mean pre-test CCRSA score was 69.02 (*SD* = 17.12) and the mean post-test CCRSA score was 76.24 (*SD* = 16.00).

A Wilcoxon signed rank test did not reveal statistically significant differences between the pre-test and post-test CADL-3 scores with a small effect size *T*= 19, *z* = .14, *p* = .89, *r* =.03. The mean pre CADL-3 score was 90.8 (*SD* = 5.28) and the mean post CADL-3 score was 89.9 (*SD* = 7.20).

### Trained Communication Measures

**Figure 3:**
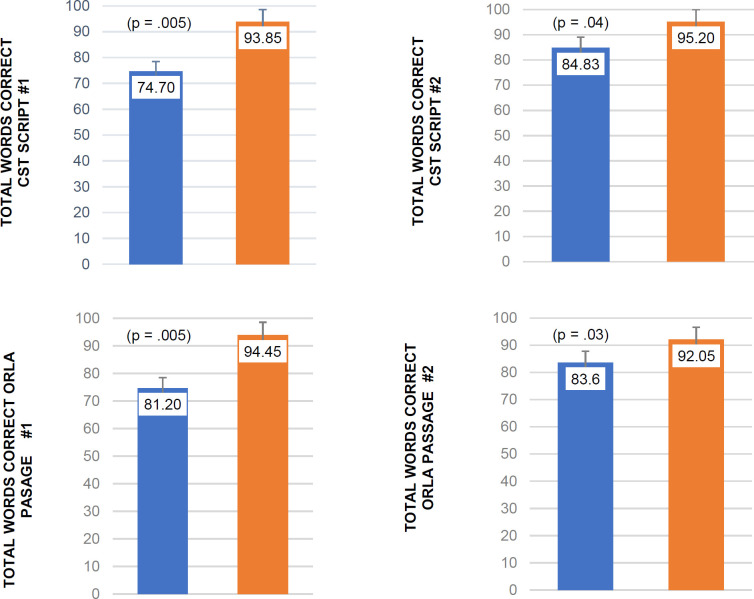
Group Means for Trained Communication Measures (CST and ORLA) Pre- and Post-Treatment

The second aim of this study was to determine if PWA would demonstrate improvements in functional communication as measured by performance after telepractice intervention on treated functional communication measures. The specific measures that were used were the total words correct for the target scripts/passages trained using CST and ORLA (see Figure 3).

#### Conversational Script Training (CST)

A Wilcoxon signed rank test revealed statistically significant improvements in the total words produced correctly for both CST scripts #1 and #2 from the initial baseline session to the final treatment session for each trained script. There were statistically significant differences between the baseline and final training session for CST script #1 with a large effect size *T* =55.00, *z* = 2.80, *p* = .005, *r* = .68. The mean baseline percentage of words produced correctly for script #1 was 74.70 (*SD* = 15.25) and the mean post-treatment percentage of words produced correctly was 93.85 (*SD* = 6.99).

Six out of ten participants mastered script #1 and proceeded to targeting script #2. A Wilcoxon signed rank test revealed significant improvements from the baseline session of CST script #2 to the last treatment session for CST script #2 with a medium effect size *T* = 15.00, *z* = 2.02, *p* = 0.04, *r* = .45. The mean baseline percentage of words produced correctly for script #2 was 84.83 (*SD* = 10.53) and the mean percentage of words produced correctly during the last treatment session was 95.20 (*SD* = 2.82).

#### Oral Reading for Language in Aphasia (ORLA)

The findings from the ORLA total words produced correctly showed statistically significant improvements between the baseline and the last treatment session for reading passages #1 and #2. All 10 participants had an increase in total words produced from the baseline of ORLA reading passage #1 to the last treatment session for the ORLA reading passage #1. A Wilcoxon signed rank test revealed significant improvements with a large effect size *T* = 55.00, *z* = 1.89, p = 0.005, *r* = .63). The mean baseline percentage of words produced correctly for passage #1 was 81.20 (*SD* = 8.78) and the mean post-treatment percentage of words produced was 94.45 (*SD* = 2.74).

A Wilcoxon signed rank test revealed significant improvements from the baseline of ORLA reading passage #2 to post-treatment ORLA reading passage #2 with a medium effect size, *T* = 27.00, *z* = 1.82, *p* = 0.03, *r* = .49. The mean baseline percentage of words produced correctly for passage #2 was 83.600 (*SD* = 14.73) and the mean percentage of words produced correctly during the last session was 92.05 (*SD* = 7.59).

### Feasibility

The third aim of this study was to determine the feasibility of telepractice for PWA as measured by a telepractice satisfaction survey. Participants' satisfaction with telepractice as an alternative service delivery model was very high. The findings also revealed high satisfaction with the use of the Zoom videoconferencing platform. The mean overall telepractice satisfaction score was 95.2 indicating the participants strongly agreed with all satisfaction components including usefulness, ease of use and learnability, interface quality, interaction quality, reliability, and satisfaction and future use. All participants (100%) agreed or strongly agreed that telepractice was useful for improving access to speech therapy services. In addition, 90% of the participants agreed or strongly agreed that the functions of the Zoom videoconferencing system were easy to learn. Most of the participants (80%) agreed or strongly agreed that the Zoom videoconferencing system was easy to understand and navigate. There was very high satisfaction with the audio and video quality with 100% of the participants agreeing or strongly agreeing that they could hear, see, and interact with the telepractice clinician as well as with an in-person clinician.

Seventy percent of the participants agreed that the Zoom videoconferencing system was reliable. Importantly, 100% of the participants strongly agreed that they would use telepractice in the future. The telepractice satisfaction results can be viewed in Table 2.

**Table 2:** Percentages for Participant Responses to Each Factor on the Telepractice Satisfaction Survey

**Answer Scale.** 1: Strongly Disagree; 2: Disagree; 3: Neither agree nor disagree; 4: Agree; 5: Strongly Agree 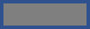 0-25%, 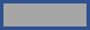 26-50%, 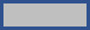 51-75%, 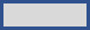 76-100%						
**Components**	**Factors**	**1** 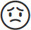	**2** 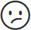	**3** 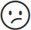	**4** 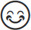	**5** 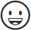
**Usefulness**						
1	Telepractice improves my access to speech therapy services.	0%	0%	0%	50%	50%
2	Telepractice saves me time traveling to a hospital or specialist clinic.	0%	0%	0%	30%	70%
3	Telepractice provides my speech therapy needs.	0%	0%	0%	20%	80%
**Ease of Use & Learnability**						
1	It was simple to use the Zoom videoconferencing system.	0%	0%	0%	20%	80%
2	It was easy to learn to use the Zoom videoconferencing system.	0%	0%	10%	10%	80%
3	I believe I could become productive quickly using the Zoom videoconferencing system.	0%	0%	10%	10%	80%
**Interface Quality**						
1	The way I interact with the Zoom videoconferencing system is pleasant.	0%	0%	0%	10%	90%
2	I like using the Zoom videoconferencing system.	0%	0%	0%	10%	90%
3	The Zoom videoconferencing system is simple and easy to understand.	0%	0%	0%	30%	70%
4	The Zoom videoconferencing system is able to do everything I would want it to be able to do.	0%	0%	20%	20%	60%
**Interaction Quality**						
1	I could easily talk to the clinician using the Zoom videoconferencing system.	0%	0%	0%	0%	100%
2	I could hear the clinician clearly using the Zoom videoconferencing system.	0%	0%	0%	10%	90%
3	I felt I was able to express myself effectively.	0%	0%	0%	20%	80%
4	Using the Zoom videoconferencing system, I can see the clinician as well as if we met in person.	0%	0%	0%	10%	90%
**Reliability**						
1	I think the visits provided via the telepractice delivery model are comparable to in-person visits.	0%	0%	10%	20%	70%
2	Whenever I made a mistake using the Zoom videoconferencing system, I could recover easily and quickly.	0%	0%	0%	40%	60%
3	The Zoom videoconferencing system gave error messages that clearly told me how to fix problems.	0%	0%	30%	10%	60%
**Satisfaction and Future Use**						
						
1	I feel comfortable communicating with the clinician using the telepractice delivery model.	0%	0%	0%	0%	100%
2	Telepractice is an acceptable way to receive therapy services.	0%	0%	0%	0%	100%
3	I would use telepractice services again.	0%	0%	0%	0%	100%
4	Overall, I am satisfied with telepractice	0%	0%	0%	0%	100%

## Discussion

The overall findings of this study suggest that telepractice is an effective and feasible alternative service delivery for SLT with PWA. While no significant differences were found in pre-versus post-treatment scores on the CADL-3, there were significant increases in CCRSA scores as well as performance on conversational scripts (i.e., CST) and reading passages (i.e., ORLA) that were trained via eight 1-hour sessions delivered via telepractice modality.

### Generalized Communication Measures

The first aim investigated if PWA would demonstrate improvements in functional communication outcome scores after telepractice intervention as measured by pre-and post-treatment language assessment scores. Very few studies have investigated the efficacy of functional language assessments and treatments administered via telehealth. A small number of studies have assessed improvements in functional communication, but very few have used standardized functional communication outcome measures (Macoir et al., 2017; Rhodes & Isaki, 2018; Simic et al., 2016). The current study expanded previous research by including functional assessment outcome measures that coincide with the Life Participation Approach to Aphasia (LPAA) service delivery model. More specifically, this study utilized the Communication Confidence Rating Scale for Aphasia (CCRSA; Cherney, Babbitt, Semik, & Heinemann, 2011) and the Communication Activities of Daily Living (CADL-3; Holland et al., 2018).

There were no significant differences in the pre-and post-treatment CADL-3 scores. Only 50% of the participants exhibited a slight numeric increase (range 1–4 points) in post-test CADL-3 scores. The fact that there were no statistically significant differences in the CADL-3 pre-test and post-test scores may be explained by the protocol allowing multimodal responses to test stimuli. Participants were allowed to respond to stimuli by answering the questions verbally and/or by using the cursor to point to the correct answers on the screen. The CADL-3 responses were scored the same regardless of the response modality. It is important to note that the CADL-3 is a functional communication assessment, and its primary clinical use is to assess functional communication skills for daily activities regardless of the modality. The CADL-3 results warrant further investigation regarding the efficacy of telepractice using other standardized functional communication measures that may be more sensitive in capturing changes in efficiency, ease, and naturalness.

The participants exhibited significant improvements in the mean pre-and-post CCRSA scores. The participants reported substantial overall increases in the total communication confidence percentage post-treatment (change in group mean =7.22). The specific areas of improvement and the degree of improvement varied across participants. However, the majority of the participants (90%) reported increased confidence in the ability to be understood when they talked to others. It is not surprising that participants demonstrated significant improvements in the CCRSA scores following CST and ORLA intervention considering the personal nature of the trained stimuli. Previous studies have found PWA have better outcomes when goals are salient and personally relevant (Cherney et al., 2015). CST and ORLA directly targeted communication topics that applied to everyday life events which may have contributed to the significant increases in communication confidence scores.

While all participants had improvements in their CCRSA scores, participants 1 and 2 exhibited the largest increase between the two time points. Participant 1 exhibited a 14-point increase and participant 2 exhibited a 17-point increase. Analysis of the participant demographics revealed participants 1 and 2 had the lowest WAB-R AQ scores out of the 10 participants in the study, indicating their aphasia was more severe than the other participants. Participant 1 had a WAB-R AQ of 71.84 and participant 2 had a WAB-R AQ of 74.4. Participants with more severe language impairments and a lower baseline for functional communication may have experienced greater perceived gains in functional communication than participants with less severe language impairments. The results were consistent with a recent meta-analysis by Kiran (2016) which found that individuals with more severe aphasia tend to show more significant gains in standardized test scores and treatment related data than those with less impairments.

Unexpectedly, one participant exhibited a decrease in the post-test CCRSA score (i.e., −6.5 points). However, the decrease could potentially be explained by environmental factors as the participant reported personal issues during the last few weeks of treatment. Environmental variables may potentially impact treatment participation, intensity, and outcomes (Harvey et al., 2020). Consistent with this report, the clinician noted distractions including answering the phone and texting during the last few sessions. While there were no statistically significant differences between the pre-and-post CADL-3, there were significant increases in the CCRSA scores. Communication confidence has been linked to a higher quality of life and positive psychosocial outcomes which coincide with the life participation approach to aphasia (Chapey et al., 2000). Therefore, it may be beneficial to measure functional communication outcomes according to the PWA and caregiver's perceived ability to communicate during activities of daily living.

### Trained Communication Measures

The second aim investigated if PWA would demonstrate improvements in functional communication as measured by performance on treated functional communication measures. It measured the improvements in expressive language skills from the baseline to the last treatment session. The participants displayed substantial improvements in the trained stimuli for both CST and ORLA. Findings from the current study are consistent with previous telepractice research studies that have found improvements in wording-finding while utilizing a variety of interventions including traditional naming treatment, phonological component analysis, and script training (Lavoie et al., 2017; Macoir et al., 2017; Rhodes & Isaki, 2018; Simic et al., 2016; Woolf et al., 2016; Zheng et al., 2016). Most similar to this study, Rhodes and Isaki, (2018) reported improvements in trained functional communication stimuli and communication confidence scores following CST delivered via telepractice. All the participants in the current study exhibited gains in CST but there was variability in the number of participants who mastered CST script #1. Six out of ten participants mastered the first script and proceeded to targeting the second script. Five of the six participants who targeted script #2 displayed numeric gains. The variability in the improvements did not appear to be correlated with the aphasia severity level.

The ORLA results revealed significant gains in the total words produced correctly. All ten participants displayed a numeric increase in the total words produced for passage #1. For passage #2, six out of ten participants exhibited numeric gains in the total words produced while four out of ten participants remained stable with no notable improvements or declines. More participants might have displayed numeric gains in the total words produced for reading passage #2 if the treatment phase had been longer. Thus, dose is an important aspect to investigate in future studies. In this study, participants received 60 minutes of SLT twice per week, which appeared to be an appropriate schedule in terms of frequency and amount of time. The participants often verbally and non-verbally indicated that they were fatigued by the end of the sessions due to the repetitive nature of the treatment. However, it might have been beneficial for the participants to receive SLT for a longer duration of 6 to 8 weeks. There are indications that increased dose yields greater improvements on language measures but there is not enough evidence to determine the optimal dose and intensity for aphasia intervention (Cherney, 2012; Harvey et al., 2020).

The findings from this study were consistent with other studies that found improvements in reading accuracy following ORLA intervention (Cherney et al., 1986; Cherney, 1995, 2010). To the researchers' knowledge, this is the only study that has implemented ORLA via synchronous telepractice modality. The participants in the current study displayed communication improvements post CST and ORLA intervention, indicating telepractice is an effective alternative service delivery model. The findings from this study contribute to the growing body of evidence suggesting telepractice is effective for improving functional communication in PWA.

**Figure 4:**
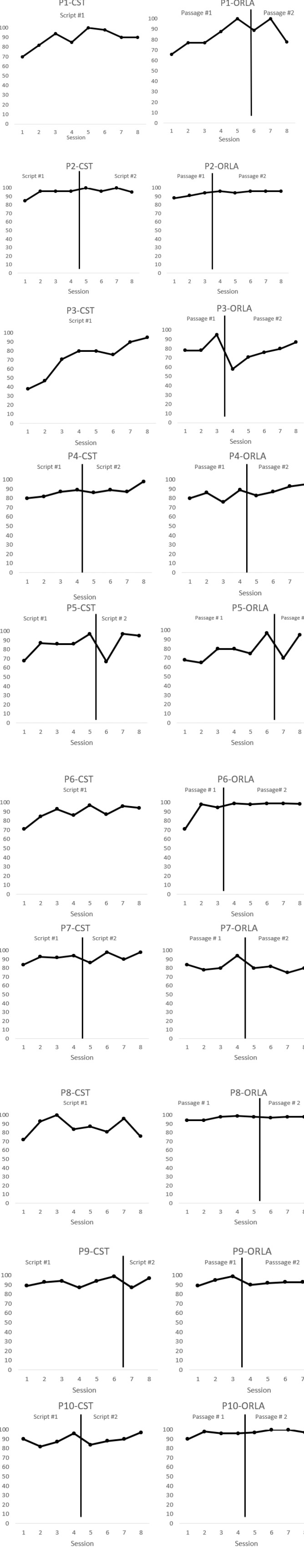
Individual Participant Treatment Data for Percentage of Total Words Correct for CST Script #1 and Script # 2 and ORLA Passage #1 and Passage # 2 Across Sessions

### Feasibility

The third aim of the study investigated the feasibility of telepractice for PWA as measured by a telepractice satisfaction survey. The objective was to provide empirical data regarding participant satisfaction with telepractice as an alternative service delivery model as well as satisfaction with the Zoom videoconferencing system. Participants' telepractice satisfaction was high in all six categories, which included the following: Usefulness, Ease of Use, Interaction Quality, Reliability, and Satisfaction/Future Use. All participants agreed or strongly agreed that telepractice was useful for improving access to speech therapy services. The satisfaction and future use of telepractice were rated very high with all participants indicating they would use telepractice in the future. The positive results are consistent with previous research indicating high patient satisfaction with telehealth (Dekhtyar et al., 2020; Guo et al., 2017; Theodoros et al., 2008). Inequitable access to SLP services among PWA has been an ongoing issue due to transportation barriers, geographical barriers, health care disparities, limited insurance reimbursement, speech-language pathologist (SLP) shortages, and case-load capacities (Choi et al., 2016; Hakim et al., 1998). Telehealth for PWA was not widely accepted prior to COVID-19. The COVID-19 pandemic has highlighted the need for an alternative service delivery model resulting in accelerated acceptance of telehealth. Patients, SLPs, caregivers, and policymakers have begun to close the divide between the perceived barriers and the true barriers of telehealth (Gustavson et al., 2021).

Telehealth has played a major role in the mitigation of access to healthcare gaps which became more evident during the COVID-19 pandemic. Although telehealth has become more widely accepted by patients and clinicians, it does not come without challenges. The COVID-19 pandemic revealed health disparities and barriers to implementing telehealth among underserved populations. Despite the increased acceptance and utilization of telehealth, many PWA continue to experience barriers due to health care disparities and low socioeconomic status. In fact, two participants in the current study received therapy at the UAMS Speech-Language and Hearing Clinic because they lacked access to a computer/tablet and internet service. Their access was further impeded by public transportation barriers. Many public bus routes were closed due to COVID-19 pandemic-related staffing shortages. In an attempt to overcome the transportation barrier that would have otherwise prevented these two individuals from participating, the study's funding provided private transportation to and from the UAMS Speech-Language and Hearing Clinic. The use of the transportation company posed other disparities including long wait times. In some instances, the participants had to wait up to four hours for the transportation company to pick them up from the clinic. There were a few instances where the private transportation company did not show up or call to cancel the ride.

### Limitations/Future Directions

Although there were several positive findings noted in the study, there were evident limitations. The small number of participants limited the ability to examine the effectiveness of the interventions across different aphasia severity levels. Seven of the ten participants exhibited mild aphasia which may have disproportionately impacted the mean outcomes. Future studies should be conducted with larger samples and a more diverse range of aphasia severity levels. Another limitation is the lack of explicit measurements to assess the generalization of gains from SLT. Future studies of telepractice treatment in PWA should investigate outcomes in various situational contexts with familiar and unfamiliar listeners during everyday activities. Future research should also utilize generalization follow-up measures to provide more robust evidence of the effectiveness of these interventions delivered via telepractice.

In addition, this study utilized a within-subjects pre-and post-test design as opposed to a randomized control trial (RCT). Changes in scores for each participant were evaluated before the interventions began and after the interventions ended. Because there was no control group of participants or evaluation of participants during a period of time when participants didn't receive therapy, changes in scores may be the result of other factors not related to intervention. However, the participants did indicate that they perceived that the interventions helped, which does add some credence to the effectiveness of the interventions. Nevertheless, future studies should consider using a control group and/or other designs that evaluate how the PWA performs during times when the interventions are not occurring. There is a need for more RCT studies to directly compare the outcomes of telepractice and in-person delivery approaches. Future studies should consider including a pre-and post-test telepractice satisfaction survey to allow the researcher to compare the perceived feasibility of telepractice pre-treatment to the perceived feasibility post-treatment. Lastly, this study may be affected by self-selection bias. The participants chose to be in this study during a time when there were unique SLT accessibility barriers caused by the ongoing COVID-19 pandemic. Moreover, it is impossible to parse out any unknown influences, such as confounding psychosocial variables that the COVID-19 pandemic might have had on the results. Nevertheless, the unprecedented times of the COVID-19 pandemic presented a unique opportunity to investigate functional telepractice outcomes. The findings from this study supported the efficacy and feasibility of ORLA and CST delivered via telepractice in PWA.

## Conclusion

In summary, this study investigated the effectiveness and feasibility of ORLA and CST via telepractice in PWA. The findings from this study were commensurate with previous studies that suggest telepractice is comparable to in-person SLT. All participants demonstrated improvements in script production during CST and reading accuracy during ORLA. They also displayed significant gains in communication confidence post-intervention. All participants were highly satisfied with telepractice as an alternative service delivery model and indicated they would use it in the future. The findings from this study and previous studies indicate telehealth is an effective and feasible alternative service delivery model for overcoming some barriers to access to speech-language treatment. However, we must continue to explore options to eliminate a myriad of accessibility barriers as telehealth becomes more widely accepted among all stakeholders.
